# Increased Calcium Supplementation Postpartum Is Associated with Breastfeeding among Chinese Mothers: Finding from Two Prospective Cohort Studies

**DOI:** 10.3390/nu8100622

**Published:** 2016-10-09

**Authors:** Jian Zhao, Yun Zhao, Colin W. Binns, Andy H. Lee

**Affiliations:** School of Public Health, Curtin University, Perth 6102, Australia; jian.zhao@postgrad.curtin.edu.au (J.Z.); c.binns@curtin.edu.au (C.W.B.); andy.lee@curtin.edu.au (A.H.L.)

**Keywords:** calcium supplementation, breastfeeding, postpartum, infant, nutrients, generalized linear mixed model, time dependent variable, pooled analysis, China

## Abstract

The calcium supplementation status during the postpartum period among Chinese lactating women is still unclear. The objective of this study is to utilize data from two population-based prospective cohort studies to examine the calcium supplementation status and to identify whether breastfeeding is associated with increased calcium supplementation among Chinese mothers after child birth. Information from 1540 mothers on breastfeeding and calcium supplementation measured at discharge, 1, 3, and 6 months postpartum were extracted to evaluate the association between breastfeeding and calcium supplementation postpartum. A generalized linear mixed model was applied to each study initially to account for the inherent correlation among repeated measurements, adjusting for socio-demographic, obstetric factors and calcium supplementation during pregnancy. In addition, breastfeeding status measured at different follow-up time points was treated as a time dependent variable in the longitudinal analysis. Furthermore, the effect sizes of the two cohort studies were pooled using fixed effect model. Based on the two cohort studies, the pooled likelihood of taking calcium supplementation postpartum among breastfeeding mothers was 4.02 times (95% confidence interval (2.30, 7.03)) higher than that of their non-breastfeeding counterparts. Dietary supplementation intervention programs targeting different subgroups should be promoted in Chinese women, given currently a wide shortage of dietary calcium intake and calcium supplementation postpartum.

## 1. Introduction

The mineral accretion rate of a neonate reaches about 30–40 mg/kg per day, while calcium transfer between mothers and infants is on average 210 mg per day [1,2,3]. For babies who are breastfed exclusively through the first 6 months, the amount of mineral demand from the mothers is four times greater than that during 9 months of pregnancy [4]. The calcium requirement of mothers during lactation has been the subject of much discussion [5,6,7]. In 2011, the Institute of Medicine published the calcium dietary reference intakes by life stage, in which Estimated Average Requirement (EAR) of calcium for pregnant and lactating adult women is recommended as 800 mg [8].

Compared to western countries, the lower consumption of dairy products in China results in that most of Chinese residents have calcium intake lower than the adequate intake (AI) [9,10,11]. In a prospective cohort study of women’s health from Shanghai, the median intake of calcium was 485 mg/day, 60% of calcium from plant sources, and only 20% from milk, which was lower than the age group specific AI (800 mg/day for 18–49 years group and 1000 mg/day for over 50 years group) [11,12]. Only 6.25% of perimenopausal women reached the standard of calcium intake in Changsha [13]. The average intake of calcium of Beijing elderly was 505 mg/day, which was about one half of the recommended adequate intake for the elderly [14]. In the National Nutrition and Health Survey of 2002, fewer than 5% reached the adequate intake levels of calcium for all age groups and the prevalence of calcium supplementation during pregnancy was 41.4% [15,16]. Besides cultural preferences, the lower consumption of dairy products in China is attributed to the high rate of lactose intolerance, which is around 80% to 95% [17,18].

The Chinese National Health and Family Planning Commission recommends that pregnant women should have a dietary calcium intake of 1000 mg per day from the second trimester and increase to 1200 mg per day from the third trimester until the end of lactation [19]. However, low dietary calcium intake in lactating women has been reported in different regions of China, as shown in Table 1. This suggests that calcium supplementation for lactating women is an important public health issue to mothers in China based on the current evidence about the benefits of calcium intake during lactation on reducing maternal bone loss [20,21,22,23].

The calcium supplementation status during postpartum period among Chinese lactating women is still unclear. The objective of the present study is to utilize data from two population-based prospective cohort studies to examine the calcium supplementation status and to identify whether breastfeeding is associated with increased calcium supplementation among Chinese mothers after child birth.

## 2. Materials and Methods

### 2.1. Study Participants

Two prospective cohort studies were conducted in an urban area, Chengdu (capital city) and a rural area, Jiangyou (county-level city), Sichuan Province, China between 2010 and 2012. Mothers who gave birth to a healthy singleton infant were invited to participate before discharge. These two studies used the same methodology based on same questionnaires, which had been used in Australia and China [29,30,31] previously, to interview all consented women face-to-face at discharge, and followed up the participants at one, three and six months postpartum by telephone interviews. The baseline interview collected detailed information on mothers and newborns, including socio-demographic, obstetric characteristics and dietary supplements during pregnancy. The follow-up interviews collected detailed information on lactation patterns and durations and dietary supplements during the postpartum period. The World Health Organization (WHO) standard definition of any breastfeeding was used in these two studies; ‘Any breastfeeding’ is defined as the infant has received breast milk (direct from the breast or expressed) with or without other drink, formula or other infant food [32].

### 2.2. Ethical Approval

The two cohort studies were approved by the Human Research Ethics Committee of Curtin University, Perth, Western Australia (approval numbers: HR169/2009 and HR168/2009, respectively). The present study was also approved by the Human Research Ethics Committee of Curtin University (approval number: RDHS-101-15). The data used in this study were de-identified.

### 2.3. Statistical Analysis

The outcome of the present study is maternal calcium supplementation status (yes or no) measured longitudinally during three different postpartum periods (from discharge to 1 month, from 1 month to 3 months, and from 3 months to 6 months, respectively) at three follow-up time points (namely, 1 month, 3 months and 6 months postpartum). The main variable of interest, any breastfeeding status, was measured longitudinally at three different postpartum time points (discharge, 1 month and 3 months postpartum). Descriptive statistics of mothers’ socio-demographic status, obstetric characteristics, calcium supplementation during pregnancy and the three postpartum periods, and any breastfeeding status at the three postpartum time points were obtained and reported. Chi-square test was conducted to compare the calcium supplementation rates between breastfeeding group and non-breastfeeding group at the different follow-up time points. Generalized linear mixed model (GLMM) was used to examine the effect of breastfeeding on calcium supplementation postpartum taking into account inherent correlations among repeated measurements. Furthermore, the breastfeeding status was included as a time-dependent variable in the longitudinal analysis. Random intercept model without covariates (Model I) was run initially to test random intercept effect, and then any breastfeeding status at the different time points and an indicator variable of measurement times were added into the above Model I to be a Model II. Furthermore, subject level socio-demographic covariates such as household annual income, maternal age and maternal education were then added into and adjusted in the Model II to formulate a Model III. Finally, obstetric characteristics such as parity, gravidity, infant gender, infant birth weight and infant gestational week, together with calcium supplementation during pregnancy, were further adjusted in the Model III to become the final Model IV. The above regression analysis was carried out for data set extracted from each cohort study separately, and the results of Model II and final Model IV were reported. In addition, a pooled effect size was calculated using a fixed effect model given that the heterogeneity between the two studies was tested being statistically nonsignificant. All statistical analyses were performed by using SAS 9.4 (SAS Institute Inc., Cary, NC, USA).

## 3. Results

### 3.1. Characteristics of Participants

For each cohort, mothers’ baseline socio-demographic status, obstetric characteristics and calcium supplementation during pregnancy are presented in Table 2. In the Jiangyou study, 695 mothers were interviewed at baseline, and 648 and 620 mothers remained in the study at 1 month and 3 months postpartum, respectively. Any breastfeeding rate dropped slightly from 93.53% at discharge to 91.05% at 1 month postpartum then continuously to 83.71% at 3 months postpartum. In the other cohort conducted in Chengdu, 845 mothers were interviewed at baseline and 760 mothers were followed up until six months postpartum. Any breastfeeding rate declined from 93.02% at discharge to 87.89% at 1 month postpartum then substantially to 73.42% at 3 months postpartum.

### 3.2. Calcium Supplementation Status during Postpartum Period

**Figure 1:**
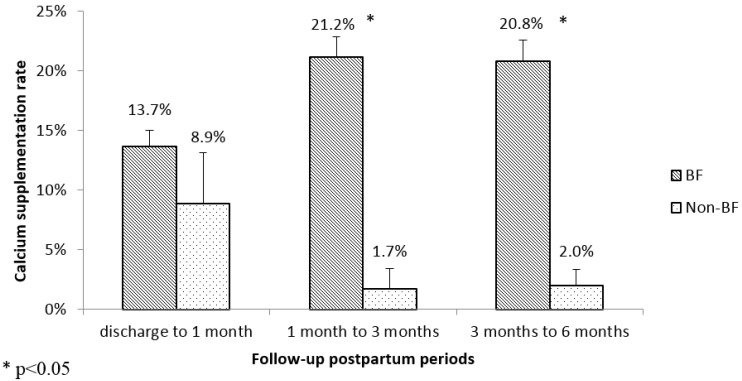
Calcium supplementation postpartum in Jiangyou.

**Figure 2:**
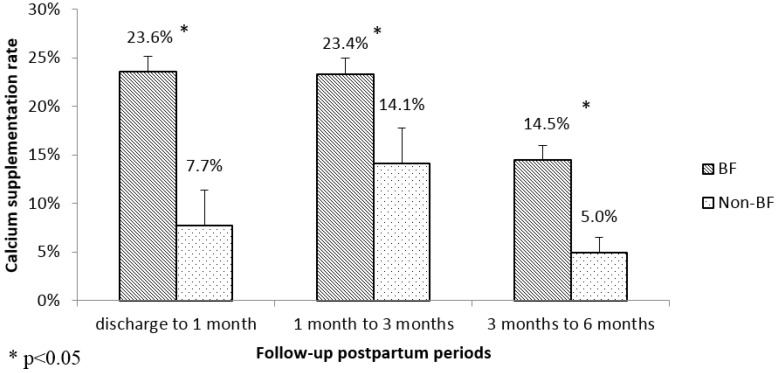
Calcium supplementation postpartum in Chengdu.

Overall, among mothers in the Jiangyou cohort, an inverted U shape of calcium supplementation rates at three different postpartum periods was observed, which corresponded to 13.4%, 19.4% and 17.7%, respectively. While in the Chengdu cohort, a constant decline trend was recorded with 22.5%, 22.2% and 12.0% reported at the three postpartum periods. When considering separately for breastfeeding and non-breastfeeding groups, as shown in Figure 1 and Figure 2, the calcium supplementation rate in the breastfeeding group was statistically significantly higher than that in the non-breastfeeding group for all the different postpartum periods, except between discharge and 1 month in the Jiangyou cohort (*p* = 0.36). In the Jiangyou cohort, calcium supplementation rates ranged from 13.7% to 21.2% for breastfeeding mothers, and ranged from 1.7% to 8.9% for non-breastfeeding mothers. In the Chengdu cohort, calcium supplementation rates reduced from around 23% in the first 3 months postpartum to 14.5% between 3 months and 6 months in breastfeeding mothers, and ranged from 5.0% to 14.1% in non-breastfeeding mothers.

### 3.3. The Association between Breastfeeding and Calcium Supplementation Postpartum

In Model I (without any covariates) for both cohorts, subject random effect was found to be statistically significant. Hence, both the primary variables of interest (i.e., breastfeeding status and the indicator variable of measurement times) were subsequently added into the Model I for examining the association between breastfeeding and calcium supplementation postpartum. As shown in Table 3, the likelihood of calcium supplementation in breastfeeding mothers were 5.85 times (95% confidence interval (CI) (2.50, 13.72)) and 2.88 times (95% CI (1.50, 5.54)) higher of that in non-breastfeeding mothers in Jiangyou and Chengdu, respectively. After adjusting for socio-demographic and obstetric factors as well as calcium supplementation during pregnancy, the odds ratios (ORs) and its 95% CI had changed appreciably to 6.95 and (2.68, 18.04) in the Jiangyou study, and 3.03 and (1.52, 6.02) in the Chengdu study, respectively. The heterogeneity between these two studies was not significant (*I*^2^ = 0.479, *p* = 0.17) statistically, therefore a fixed effect model was used to pool the ORs of the two studies. The pooled analysis of these two cohort studies revealed that calcium supplementation postpartum was significantly positively associated with breastfeeding with an adjusted OR = 4.02 with a 95% CI of (2.30, 7.03).

## 4. Discussion

To our knowledge, the present study is the first population-based study that determines the longitudinal trend of calcium supplementation by Chinese women from discharge to 6 months postpartum and the effect of breastfeeding on calcium supplementation. A relatively low level of calcium supplementation (less than 23%) was observed throughout the postpartum period in either breastfeeding mothers or non-breastfeeding mothers. The pooled effect size after adjusting for socio-demographics variables (household annual income, maternal age and maternal education); obstetric factors (parity, gravidity, infant gender, infant birth weight and infant gestational week); and calcium supplementation during pregnancy reveals that mothers who breastfed their babies were 4.02 times more likely to take calcium supplements compared to their non-breastfeeding counterparts during postpartum. The present result is consistent with previous findings that breastfeeding mothers consumed more calcium than non-breastfeeding counterparts [33,34,35]. One reason leading to the higher calcium supplementation in breastfeeding mothers may be the general belief that adequate calcium intake is beneficial to breast milk production, and mothers’ special attention to infants’ calcium intake under the context of wide shortage of calcium intake for Chinese women, in spite of recent evidence demonstrating that calcium supplementation in lactation has no significant effect on increasing calcium content in breast milk [36,37,38]. The other reason might be mothers’ perception of the beneficial effect of calcium supplementation on maternal bone loss during lactating. Some studies found little benefits of calcium supplementation on maternal bone loss during lactating [36,39,40], whereas other studies carried out in the U.S. and Brazil suggested that higher calcium intake during early lactation could minimize the bone loss for the mothers who had daily calcium intake less than 500 mg [20,21]. Further investigation on the factors contributing to difference of calcium supplementation between breastfeeding mothers and non-breastfeeding mothers as well as the effect of calcium supplementation on reducing maternal bone loss during lactation or enhancing maternal skeleton remodeling and remineralization after weaning of breastfeeding is recommended.

Given the habitually lower calcium dietary intake and relatively high lactose intolerance rate in the general Chinese population [12,15,17], calcium supplementation plays an important role on bone health, especially for exclusive breastfeeding women who provide around 300 mg of calcium per day to their babies via breast milk which accompany maternal bone calcium turnover [41].

This study had several strengths. We utilized data from two cohort studies to investigate the longitudinal trends of calcium supplementation at three different postpartum time points (i.e., 1 month, 3 months and 6 months postpartum) and conducted random effect regression modelling accounting for inherent dependency between the repeated measurements. Moreover, since the breastfeeding status was measured longitudinally as well in two cohorts, it was treated as a time-dependent variable in the analysis to account for possible feedback effects between the breastfeeding status and calcium supplementation at different times. In addition, our pooled analysis based on the two individual studies yielded the combined effect size with a larger sample size and higher statistical power. Moreover, calcium supplementation during pregnancy was adjusted in the modelling to control for the consequent effect of calcium intake during pregnancy on calcium supplementation during lactation.

A caveat of this study was that both cohort studies were carried out in Sichuan Province, which may limit the results being able to generalize to other regions of China. Sichuan Basin has special geographic characteristics, where the number of cloudy or rainy days is substantially larger than that in other regions in China, which may lead to a relatively lower level of vitamin D synthesis and calcium deficiency consequently [42]. However, to the best of our knowledge, no data were available currently on calcium supplementation during postpartum in other regions of China for comparison purpose.

## 5. Conclusions

In conclusion, calcium supplementation during postpartum in Sichuan is variable at different times postpartum with a relatively low level (less than 23%). Although breastfeeding has a substantive effect on calcium supplementation postpartum, dietary supplementation intervention programs and health education targeting different subgroups (e.g., breastfeeding mothers and bottle feeding mothers) should be promoted in Chinese women, given currently a wide shortage of dietary calcium intake and calcium supplementation during postpartum.

## Figures and Tables

**Table 1:** Dietary calcium intake of lactating women in different regions of China.

Study Location	Study Design	Study Period	Average Daily Dietary Calcium Intake (Postpartum)
Guangzhou [24]	Prospective cohort	2002	786.45 mg (12 weeks)
Hunan [25]	Cross-sectional	2011–2012	426 mg
Beijing, Suzhou & Guangzhou [26]	Cross-sectional	2011–2012	401.4 mg (0–1 month)
			585.3 mg (1–2 months)
			591.2 mg (2–4 months)
			649.0 mg (4–8 months)
Fujian [27]	Prospective cohort	2012	428 mg (2 days)
			454 mg (7 days)
			595 mg (30 days)
			544 mg (90 days)
Shanghai [28]	Prospective cohort	2014–2015	749.3 mg (1–3 days)
			781.1 mg (7–9 days)
			762.3 mg (14–17 days)
			768.4 mg (25–27 days)
			678.5 mg (39–41 days)

**Table 2:** Characteristics of participants at baseline by breastfeeding status.

Variable	Cohort in Jiangyou (*n* = 695)		Cohort in Chengdu (*n* = 845)	
	BF	Non-BF	BF	Non-BF
Number of participants	650 (93.5)	45 (6.5)	786 (93.0)	59 (7.0)
**Household annual income (Chinese yuan)**				
<2000	186 (31.0)	9 (23.1)	1 (0.2)	0 (0.0)
2000–5000	309 (51.4)	23 (59.0)	155 (23.5)	12 (24.0)
>5000	106 (17.6)	7 (17.9)	503 (76.3)	38 (76.0)
**Maternal age (years)**				
<25	373 (57.4)	26 (57.8)	156 (19.9)	5 (8.5)
25–29	163 (25.1)	13 (28.9)	372 (47.3)	28 (47.5)
>29	114 (17.5)	6 (13.3)	258 (32.8)	26 (44.0)
**Maternal education**				
Secondary school or lower	355 (54.6)	25 (55.6)	90 (11.5)	11 (18.6)
Senior school	215 (33.1)	18 (40.0)	165 (21.0)	11 (18.6)
University or higher	80 (12.3)	2 (4.4)	531 (67.5)	37 (62.8)
**Parity**				
Primiparous	518 (79.7)	37 (82.2)	700 (89.1)	51 (86.4)
Multiparous	132 (20.3)	8 (17.8)	86 (10.9)	8 (13.6)
**Gravidity**				
Primigravida	249 (38.3)	18 (40.0)	430 (54.7)	26 (44.1)
Multigravida	401 (61.7)	27 (60.0)	356 (45.3)	33 (55.9)
**Infant gender**				
Male	328 (50.5)	26 (57.8)	412 (52.4)	34 (57.6)
Female	322 (49.5)	19 (42.2)	374 (47.6)	25 (42.4)
**Infant birth weight (g)**				
<2500	10 (1.5)	2 (4.4)	13 (1.7)	0 (0.0)
≥2500	640 (98.5)	43 (95.6)	773 (98.3)	59 (100.0)
**Infant gestational week**				
<37	8 (1.2)	3 (6.8)	9 (1.2)	2 (3.4)
≥37	640 (98.8)	41 (93.2)	777 (98.8)	57 (96.6)
**Calcium supplementation during pregnancy**				
Yes	410 (63.1)	25 (55.6)	627 (79.8)	47 (79.7)
No	240 (36.9)	20 (44.4)	159 (20.2)	12 (20.3)

Data are presented as *n* (%); BF: any breastfeeding; Non-BF: non-breastfeeding.

**Table 3:** Association between breastfeeding status and calcium supplementation postpartum.

Variable	Model II	Model IV
	Crude ORs (95% CI)	Adjusted ORs (95% CI)
**Jiangyou Cohort**		
Measurement times *		
At discharge (ref)	1	1
1 month	1.72 (1.24, 2.38)	1.90 (1.33, 2.70)
3 months	1.57 (1.12, 2.20)	1.69 (1.18, 2.44)
Breastfeeding status *		
Non-breastfeeding (ref)	1	1
Any breastfeeding	5.85 (2.50, 13.72)	6.95 (2.68, 18.04)
**Chengdu Cohort**		
Measurement times *		
At discharge (ref)	1	1
1 month	1.02 (0.74, 1.43)	1.02 (0.72, 1.45)
3 months	0.31 (0.21, 0.46)	0.30 (0.20, 0.45)
Breastfeeding status *		
Non-breastfeeding (ref)	1	1
Any breastfeeding	2.88 (1.50, 5.54)	3.03 (1.52, 6.02)
**Pooled effect size of two studies**		
Non-breastfeeding (ref)	-	1
Any breastfeeding	-	4.02 (2.30, 7.03)

Crude ORs (obtained from Model II): Model included breastfeeding status and the indicator variable of measurement times; Adjusted ORs (obtained from the final Model IV): Model adjusted for socio-demographics variables (household annual income, maternal age and maternal education); obstetric factors (parity, gravidity, infant gender, infant birth weight and infant gestational week); and calcium supplementation during pregnancy; * *p* < 0.05; ref: reference category.
